# Human T cells efficiently control RSV infection

**DOI:** 10.1172/jci.insight.168110

**Published:** 2023-06-08

**Authors:** Chandrav De, Raymond J. Pickles, Wenbo Yao, Baolin Liao, Allison Boone, Mingyu Choi, Diana M. Battaglia, Frederic B. Askin, Jason K. Whitmire, Guido Silvestri, J. Victor Garcia, Angela Wahl

**Affiliations:** 1 International Center for the Advancement of Translational Science,; 2 Division of Infectious Diseases, Department of Medicine,; 3 Center for AIDS Research,; 4 Department of Microbiology and Immunology, and; 5 Marsico Lung Institute, University of North Carolina (UNC) at Chapel Hill, Chapel Hill, North Carolina, USA.; 6 Department of Infectious Diseases, Guangzhou Eighth People’s Hospital, Guangzhou Medical University, Guangzhou, China.; 7 Department of Pathology,; 8 Department of Genetics, and; 9 Lineberger Comprehensive Cancer Center, UNC at Chapel Hill, Chapel Hill, North Carolina, USA.; 10 Yerkes National Primate Research Center, Emory University, Atlanta, Georgia, USA.; 11 Department of Pathology and Laboratory Medicine, Emory University School of Medicine, Atlanta, Georgia, USA.

**Keywords:** Immunology, Virology, Adaptive immunity, Mouse models, T cells

## Abstract

Respiratory syncytial virus (RSV) infection causes significant morbidity and mortality in infants, immunocompromised individuals, and older individuals. There is an urgent need for effective antivirals and vaccines for high-risk individuals. We used 2 complementary in vivo models to analyze RSV-associated human lung pathology and human immune correlates of protection. RSV infection resulted in widespread human lung epithelial damage, a proinflammatory innate immune response, and elicited a natural adaptive human immune response that conferred protective immunity. We demonstrated a key role for human T cells in controlling RSV infection. Specifically, primed human CD8^+^ T cells or CD4^+^ T cells effectively and independently control RSV replication in human lung tissue in the absence of an RSV-specific antibody response. These preclinical data support the development of RSV vaccines, which also elicit effective T cell responses to improve RSV vaccine efficacy.

## Introduction

Respiratory syncytial virus (RSV), a negative-sense single-stranded RNA virus is the most common cause of acute lower respiratory tract infections in infants. Globally, RSV infection is associated with more than 100,000 deaths in infants and young children annually (1, 2). RSV is also a substantial cause of morbidity and mortality in the elderly, with approximately 200,000 hospitalizations and 14,000 deaths attributable to RSV infection in adults ≥ 65 years old in the United States (3). RSV is a significant threat to immunosuppressed individuals, including transplant recipients. Mortality rates are increased in this population compared with RSV-infected immunocompetent individuals (4–6). The COVID-19 pandemic disrupted the seasonality and intensity of RSV epidemics resulting in recent surges of RSV cases in young children. These unprecedented RSV surges have increased RSV-related hospitalizations, and the triple threat of influenza virus, RSV, and COVID-19 infections has exacerbated the burden of respiratory viruses on health care (7).

Currently, 2 therapeutics are approved to treat RSV infection: a monoclonal antibody, palivizumab (Synagis), and the antiviral nucleoside analog, ribavirin. Palivizumab is used prophylactically for preventing severe RSV infections in high-risk children, and ribavirin is used to limit the severity of established RSV infections (8–10). The single-dose long-acting monoclonal antibody niresivamib was also recently approved by the European Union for RSV prevention in infants. Additional RSV antivirals have been developed, but despite promising preclinical efficacy, they failed to achieve efficacy and safety in larger clinical trials (9, 10). There is a major need for an RSV vaccine, especially for infants and young children. Although vaccine candidates for RSV are in advanced clinical development, currently no licensed RSV vaccine is available worldwide, in part due to the lack of well-defined correlates of protection against RSV infection (11). Candidate RSV vaccines in clinical development — including those recently evaluated by Pfizer, Moderna, and GlaxoSmithKline, which have shown efficacy against RSV-associated lower respiratory tract disease in infants of vaccinated mothers and/or older individuals in phase III clinical trials — are primarily designed to elicit neutralizing antibodies to the prefusion conformation of the RSV Fusion (F) protein responsible for virus penetration into host cells (12–15). The variable clinical efficacy of monoclonal antibodies targeting the RSV F protein to prevent RSV infection highlights the need for targeting multiple antigenic sites on the RSV F protein due to genetic variability at these sites in circulating strains (16). Some vaccine approaches (e.g., particle-based and vector-based vaccines) also induced RSV-specific cell-mediated immune responses in clinical trials (12). While a vaccine-induced RSV-specific T cell response would not be able to prevent infection, it could accelerate virus clearance and ameliorate disease if vaccine-elicited antibodies fail to prevent infection. The implementation of in vivo models of RSV infection that recapitulate the outcomes and immune responses that occur during human RSV infection are highly desirable for testing the efficacy of RSV vaccine candidates (17).

We previously demonstrated that, following implantation of human lung tissue into immunodeficient mice (lung-only mice [LoM]), the human lung tissue continues to develop over time, with human epithelial cells, smooth muscle cells, fibroblasts, and endothelial cells forming human lung structures with extensive vascularization (18, 19). Specifically, human lung implants maintain the structural and cellular architecture of the human cartilaginous conducting airways, terminal bronchiolar airways, and distal alveolar regions, providing an in vivo model for investigating bacterial and viral infections in the human lung (18, 19). Our previous studies demonstrated the utility of the human lung implants for investigating human respiratory virus infections without requiring species adaption, including the recently emerged coronaviruses SARS-CoV, Middle East Respiratory Syndrome–CoV (MERS-CoV), and SARS-CoV-2 (18, 19). When LoM are also reconstituted with autologous HLA-matched human innate and adaptive immune cells (creating bone marrow/liver/thymus–lung [BLT-L] mice), human lung infections elicit pathogen-specific human antibody and T cell responses that control virus replication (e.g., human cytomegalovirus [HCMV]) (18). Our aim was to use these complementary in vivo models (LoM and BLT-L mice) to evaluate human immune correlates of protection from RSV infection, with an emphasis on the role of human T cells in mediating virus clearance in lung tissue.

Here, we show that, in the absence of an autologous human immune system (LoM), RSV infection was sustained over time and not cleared. In contrast, in BLT-L mice, RSV infection induced a virus-specific human antibody (IgM and IgG) and T cell response capable of preventing infection following a second RSV challenge. Importantly, we show that primed human CD8^+^ T cells — and, to a lesser extent, human CD4^+^ T cells — can effectively and independently control RSV infection in human lung tissue in the absence of an RSV-specific antibody response. Together, these preclinical data support an important path forward and provide in vivo models toward the development and testing of RSV vaccines that elicit protective T cell responses.

## Results

### RSV infection of LoM is robust and sustained.

**Figure 1:**
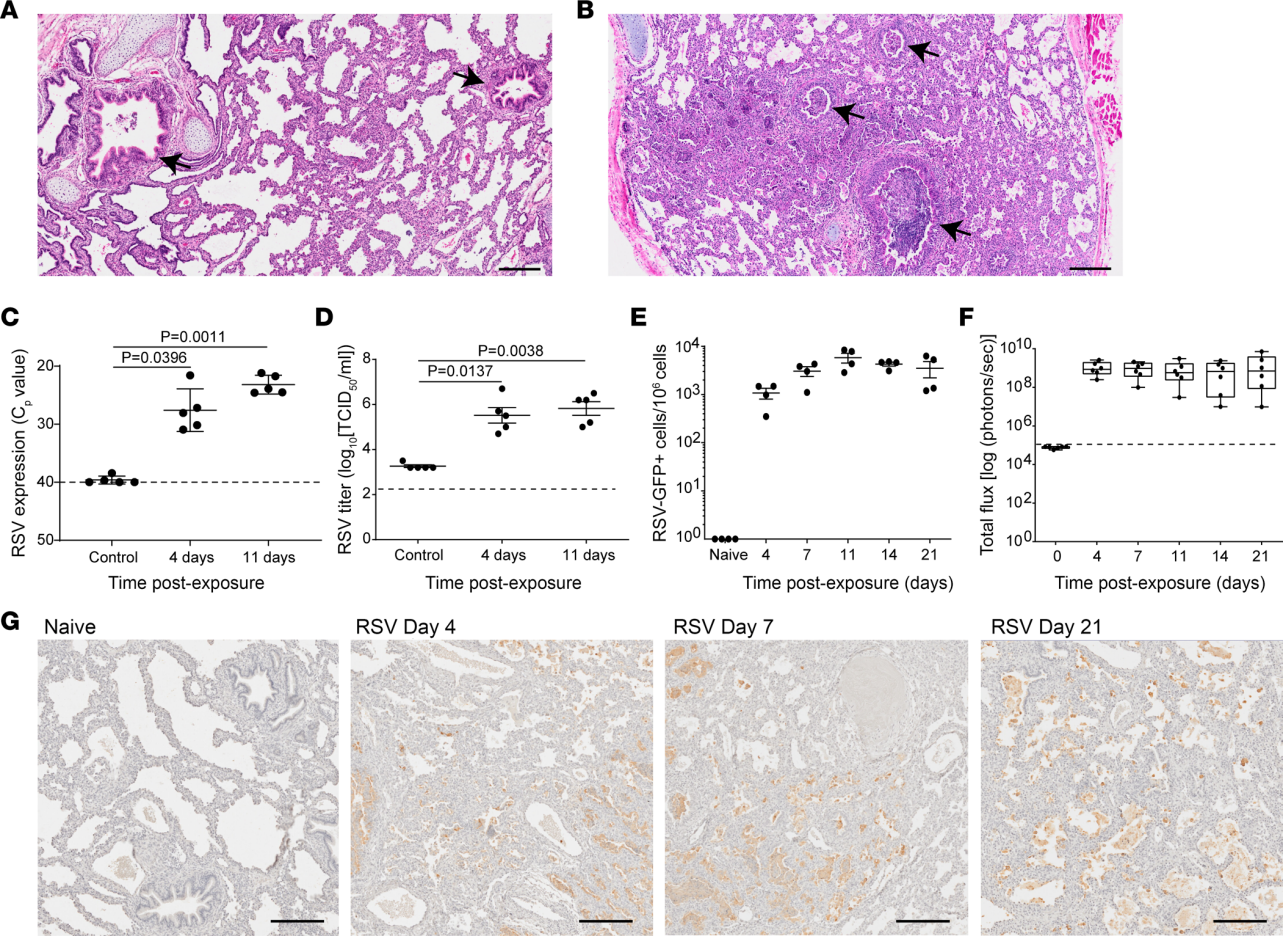
Sustained replication of RSV in human lung implants. (**A** and **B**) H&E staining of (**A**) a naive LoM human lung implant (*n* = 5 implants analyzed) and (**B**) a human lung implant from an RSV-infected LoM (*n* = 12 implants analyzed). Scale bars: 200 μm. Arrows note airways. (**C**) RSV-RNA expression in the human lung implants of control (2 hours after RSV exposure) and RSV-infected LoM at 4 and 11 days after exposure (*n* = 5 implants/time point). Crossing point (Cp) indicates the cycle number at which the fluorescence signal of the sample exceeds a background fluorescence value. (**D**) RSV titers (log_10_TCID_50_/mL) in human lung implants of control LoM (2 hours after RSV exposure) and RSV-infected LoM at 4 days and 11 days after RSV exposure (*n* = 5 implants/time point). Dashed line indicates the assay limit of detection. (**E**) Number of GFP^+^ cells as determined by flow cytometry in the human lung implants of naive LoM (*n* = 4 implants) and RSV A2-GFP–infected LoM 4, 7, 11, 14, and 21 days after exposure (*n* = 4 implants/time point). (**F**) RSV replication monitored longitudinally in the human lung implants of RSV-Luc infected LoM (*n* = 6 implants) as measured by bioluminescence (radiance [p sec^–1^ cm^–2^ sr^–1^] represented as total flux) signal. The median (horizontal line), upper and lower quartiles (box ends), and minimum to maximum values (whiskers) are shown. Background luminescence (day 0) is denoted by the dashed line. (**G**) IHC staining for RSV antigen in the human lung implants of naive LoM (*n* = 4 implants analyzed) and RSV-infected LoM 4, 7, and 21 days after exposure (positive cells are brown, *n* = 4 implants analyzed/time point). Scale bars: 200 μm. (**C**–**E**) Data are shown as the mean ± SEM; (**C** and **D**) a statistical analysis was performed using a 2-tailed Kruskal-Wallis test. *P* values were adjusted for multiple testing using the Benjamini, Krieger, and Yekutieli FDR method.

Human lung implants in LoM were inoculated with RSV as previously described, resulting in infection of human epithelial cells in lung implants (18). Gross histopathology of RSV-infected human lung implants showed robust consolidation of the lung tissue, including extensive obstruction of the airway lumens reminiscent of autopsy lung tissues from human RSV infections (Figure 1, A and B) (20, 21). To assess the extent and duration of RSV infection in LoM, we evaluated the RSV viral load at 2 hours after inoculation (controls), as a measurement of the virus inoculum, and at 4 days and 11 days after inoculation (Figure 1, C and D). RSV-RNA abundance in human lung implants increased over time and was significantly higher at 4 days (*P* = 0.0396) and 11 days (*P* = 0.0011) after inoculation compared with controls (Figure 1C). Virus titers also increased over time and were more than 2 logs higher at 4 days (5.52 log_10_ 50% tissue culture infectious dose [TCID_50_]/mL, *P* = 0.0137) and 11 days (5.82 log_10_[TCID_50_/mL], *P* = 0.0038) after inoculation compared with controls (3.26 log_10_[TCID_50_/mL]) (Figure 1D).

To quantify the numbers of RSV-infected cells, implants were inoculated with RSV strain A2 containing GFP (RSV A2-GFP) (22), harvested at specific time points, and the number of GFP^+^ cells was quantified by flow cytometry. In contrast to naive control LoM, which contained no GFP^+^ cells, GFP^+^ cells were readily detected in human lung implants at 4 days after inoculation with 1,070 ± 290 GFP^+^ cells/1 ***×*** 10^6^ live cells present (Figure 1E and Supplemental Figure 1A; supplemental material available online with this article; https://doi.org/10.1172/jci.insight.168110DS1). Over time, the numbers of GFP^+^ cells increased with 3,060 ± 700 GFP^+^ cells/1 ***×*** 10^6^ live cells and 5,830 ± 330 GFP^+^ cells/1 ***×*** 10^6^ live cells at 7 and 11 days after inoculation, respectively (Figure 1E). The numbers of RSV-infected GFP^+^ cells were then maintained until at least 21 days after inoculation — the last time point assessed (Figure 1E). RSV-infected GFP^+^ cells were not detected in the mouse lungs of animals containing RSV-infected human lung implants (Supplemental Figure 1B). We also used recombinant RSV-expressing luciferase (RSV A2-Luc) to perform noninvasive serial monitoring of RSV infection over time (23). In agreement with the results described above, luciferase expression was increased at 4 days after inoculation and was maintained over time in the lung implants of LoM (Figure 1F and Supplemental Figure 1C).

RSV antigen staining was robust and abundantly distributed throughout LoM human lung implants at 4 days after RSV inoculation (Figure 1G and Supplemental Figure 1A). RSV antigen continued to be detected in LoM harvested at 7 days and 21 days after inoculation (Figure 1G). RSV antigen was not detected in the mouse lungs of animals containing RSV-infected lung implants (Supplemental Figure 1B). Together, these data demonstrate that the human lung implants of LoM fully support robust RSV replication, which is maintained over time.

### RSV induces an innate immune response characterized by the production of proinflammatory chemoattractants and neutrophil recruitment.

**Figure 2:**
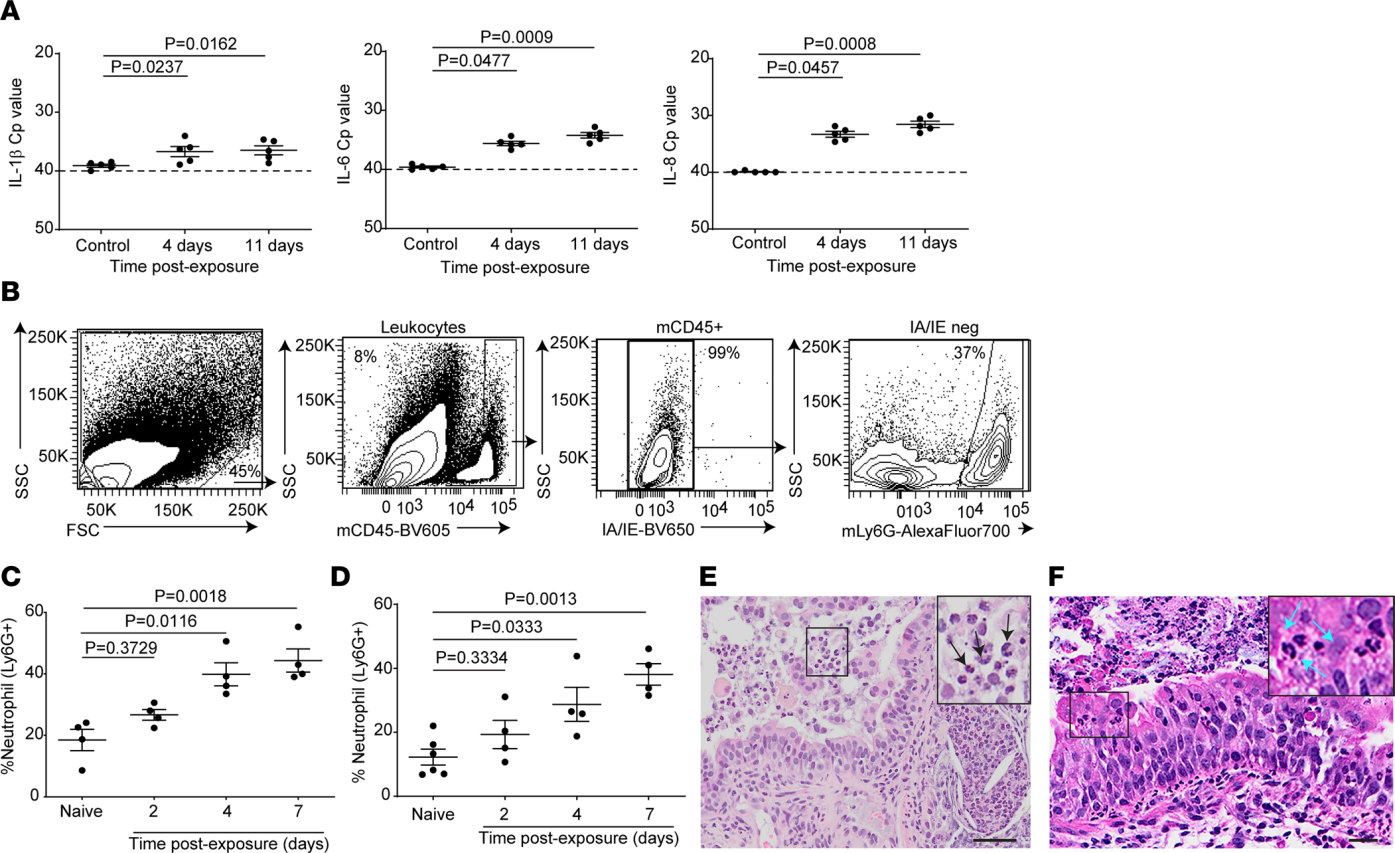
RSV infection induces an innate proinflammatory immune response resulting in neutrophil infiltration. (**A**) Human IL-1β (left panel), IL-6 (middle panel), and IL-8 (right panel) expression in the human lung implants of control LoM (2 hours after RSV exposure) and RSV-infected LoM at 4 days and 11 days after exposure (*n* = 5 implants/time point). Crossing point (Cp) indicates the cycle number at which the fluorescence signal of the sample exceeds a background fluorescence value. (**B**) Flow cytometry gating strategy used to detect neutrophils. (**C** and **D**) Levels of mouse neutrophil (%Ly6G^+^ of mouse CD45^+^ cells) in the human lung implants of 2 different human donor cohorts of naive LoM (*n* = 4 and 6 implants) and RSV-infected LoM 2 days, 4 days, and 7 days after exposure (*n* = 4 implants/time point/cohort). (**E** and **F**) H&E staining of an RSV-infected LoM human lung implant depicting (**E**) neutrophil accumulation in airway lumen (arrows; scale bars: 50 μm) and (**F**) neutrophil transmigration (arrows; scale bars: 20 μm). (**A**, **C**, and **D**) Data are shown as mean ± SEM. Statistical significance was determined with a 2-tailed Kruskal-Wallis test. *P* values were adjusted for multiple testing using the Benjamini, Krieger, and Yekutieli FDR method.

RSV bronchiolitis is highly associated with robust neutrophil recruitment (24–26). To determine whether neutrophil chemoattractants were generated by RSV-infected human lung implants, we compared human IL-1β, IL-6, and IL-8 mRNA levels present in control animals with those at days 4 and 11 after RSV exposure. Significantly higher mRNA levels of IL-1β, IL-6, and IL-8 were measured at day 4 (IL-1β, *P* = 0.0237; IL-6, *P* = 0.0477; IL-8, *P* = 0.0457) and day 11 (IL-1β, *P* = 0.0162; IL-6, *P* = 0.0009; IL-8, *P* = 0.0008) after RSV exposure (Figure 2A). Neutrophil recruitment to RSV-infected lung implants was examined by quantifying the neutrophil levels in the lung implants of naive LoM and RSV-infected LoM at days 2, 4 and 7 after exposure using flow cytometry. Significantly higher neutrophil levels were present in RSV-infected LoM at day 4 (39.85% ± 3.76% [mean ± SEM] of CD45^+^ cells; *P* = 0.0116) and day 7 (44.32% ± 3.75% [mean ± SEM] of CD45^+^ cells; *P* = 0.0018) after RSV exposure compared with naive controls (18.5% ± 3.5% [mean ± SEM] of CD45^+^ cells) (Figure 2, B and C). These results were confirmed in a separate experiment using LoM from a different donor (Figure 2D). In histological sections of RSV-infected lung implants, neutrophils were seen transmigrating across the airway epithelium and accumulating in the airway lumen (Figure 2, E and F). Neutrophil transmigration across the airway epithelium was accompanied by an abundance of necrotic cellular material accumulating in the airway lumen (Figure 2F). These data indicate that RSV induced an innate immune response characterized by the production of proinflammatory chemoattractants resulting in neutrophil recruitment to the site of infection.

### RSV infection is effectively controlled by the human immune system in BLT-L mice.

**Figure 3:**
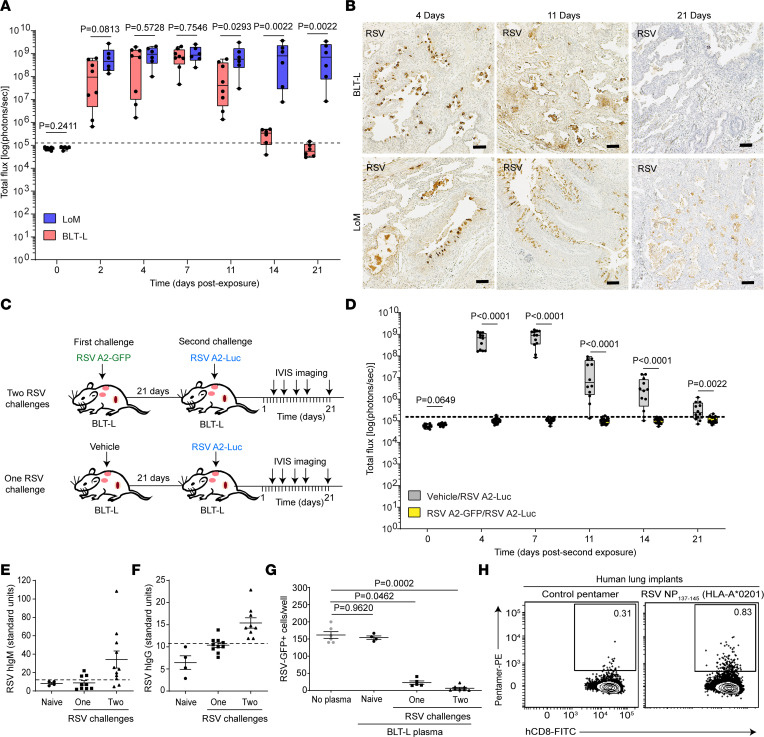
RSV infection is efficiently controlled by the human immune system in BLT-L mice and elicits protective immunity from a second RSV challenge. (**A**) Bioluminescence (radiance [p sec^–1^ cm^–2^ sr^–1^] represented as total flux) in human lung implants of RSV A2-Luc exposed LoM (blue bars, *n* = 6 implants) and BLT-L mice (red bars, *n* = 8 implants). Dashed line indicates background (preexposure) luminescence. (**B**) RSV antigen (brown) in LoM (*n* = 4 implants analyzed/time point) and BLT-L mouse (*n* = 4 implants analyzed/time point) lung implants on days 4, 11, and 21 after exposure. Scale bars: 100 μm. (**C**) BLT-L mouse lung implants were inoculated with RSV A2-GFP or vehicle (first exposure) and then RSV A2-Luc 21 days later (second exposure). (**D**) Bioluminescence signal in lung implants of RSV A2-Luc–exposed BLT-L mice that were exposed first to RSV A2-GFP (*n* = 6 implants; yellow bars) or vehicle (*n* = 12 implants; gray bars). The median (horizontal line), upper and lower quartiles (box ends), and minimum to maximum values (whiskers) are shown. Dashed line indicates background luminescence (day 0). (**E** and **F**) RSV-specific human (**E**) IgM and (**F**) IgG plasma levels in naive BLT-L mice (*n* = 4) and BLT-L mice exposed once (IgM, *n* = 9; IgG, *n* = 10) or twice (IgM, *n* = 11; IgG, *n* = 9) to RSV. Dashed line indicates seropositivity threshold. (**G**) RSV neutralization activity of plasma from naive BLT mice (*n* = 4) and BLT mice exposed once (*n* = 5) or twice (*n* = 8) to RSV. Shown is the number of RSV GFP^+^ cells 72 hours after infection representative of 6 replicates. (**H**) RSV NP_137-145_ pentamer-reactive CD8^+^ T cells in the lung implant of a BLT-L mouse exposed twice to RSV. (**E**–**G**) Data are shown as mean ± SEM. Two-tailed (**A** and **D**) Mann-Whitney *U*, and (**G**) Kruskal-Wallis tests were used to determine statistical significance. *P* values were adjusted for multiple testing using the Benjamini, Krieger, and Yekutieli FDR method.

BLT-L humanized mice are the only animal model that is systemically reconstituted with human innate and adaptive immune cells and is implanted with autologous human lung tissue, allowing for the induction of HLA-restricted pathogen-specific human antibody and T cell responses (18). LoM and BLT-L mice were inoculated with RSV A2-Luc, and infection was monitored longitudinally by luciferase bioluminescence. RSV-Luc bioluminescence was detectable in LoM and BLT-L mouse human lung implants as early as day 2 after inoculation (Figure 3A). In LoM, RSV infection was sustained up to 21 days after inoculation — the last time point analyzed (Figure 3A). In contrast, by 11 days after inoculation, RSV-induced bioluminescence was significantly lower in BLT-L mice (1.7 × 10^8^ ± 8.1 × 10^7^ [mean ± SEM] radiance, *P* = 0.0293) compared with LoM (9.7 × 10^8^ ± 4.6 × 10^8^ [mean ± SEM] radiance) (Figure 3A). While RSV infection in LoM was sustained over time, bioluminescence in BLT-L mice continued to decline and was reduced to background levels by 21 days after inoculation (Figure 3A). Similarly, abundant RSV antigen was observed at 4 and 11 days after inoculation in LoM and BLT-L mice, confirming efficient infection of lung implants in both models; however, no RSV antigen was readily detected in BLT-L mice at 21 days after inoculation, indicating effective clearance of RSV-infected cells in BLT-L mice (Figure 3B). Together, using 2 independent measures of RSV infection, these results demonstrate that human immune cells present in BLT-L mice can efficiently clear RSV infection in vivo.

To determine if BLT-L mice are protected against a second RSV challenge after clearing a primary RSV infection, human lung implants of BLT-L mice were inoculated with either RSV A2-GFP or vehicle control (mock infected); 21 days later, when primary infection was fully controlled, the lung implants from the same animals were challenged with RSV A2-Luc (Figure 3C). In BLT-L mice first inoculated with vehicle and then challenged with RSV-Luc, the kinetics of RSV infection were similar to those observed during primary RSV infection of BLT-L mice (Figure 3D). In stark contrast, no RSV infection, measured as luciferase activity, was detected in BLT-L mice previously challenged with RSV A2-GFP (Figure 3D). Specifically, the bioluminescence signal in lung implants remained at background levels throughout the course of the experiment (Figure 3D).

In separate experiments, we took advantage of the fact that BLT-L mice have 2 human lung implants (left and right) to investigate if the protective effect of a primary RSV challenge is restricted to the inoculated lung implant or due to systemic immunity affecting both lung implants in a single mouse. The left lung implant of BLT-L mice was inoculated with RSV A2-Luc, and RSV infection was monitored for 21 days in both the left and right lung implants of mice (Supplemental Figure 2A). In the left lung implant, RSV infection peaked at day 7 after inoculation and was cleared by 21 days after inoculation (Supplemental Figure 2B). In contrast, RSV-induced bioluminescence was not detected in the contralateral (right) lung implants of these mice at any time point during the 21-day analysis (Supplemental Figure 2B). Subsequently, the right lung implant of these mice was then inoculated with RSV A2-Luc (Supplemental Figure 2B). When the right lung implants were challenged with RSV, RSV infection was not detected in either of the lung implants (Supplemental Figure 2B), indicating that primary RSV infection confers protective systemic immunity against a subsequent RSV challenge.

### RSV infection elicits human neutralizing antibodies in BLT-L mice.

RSV-specific human IgM and IgG levels were measured in the plasma of BLT-L mice following RSV inoculation and detected in 33% and 30% of mice, respectively (Figure 3, E and F). RSV-specific IgM and IgG were detected more frequently in the plasma of mice challenged twice with RSV (82% [*P* = 0.0300] and 100% [*P* = 0.0031], respectively) (Figure 3, E and F). Plasma samples collected from BLT-L mice challenged once or twice with RSV had neutralizing activity, resulting in a significant reduction in RSV infection in vitro (*P* = 0.0462 and *P* = 0.0002, respectively) (Figure 3G). These results demonstrate that RSV infection of BLT-L mice results in the production of neutralizing human antibodies against RSV.

### Human CD8^+^ T cell depletion impairs but does not eliminate control of RSV infection in BLT-L mice.

**Figure 4:**
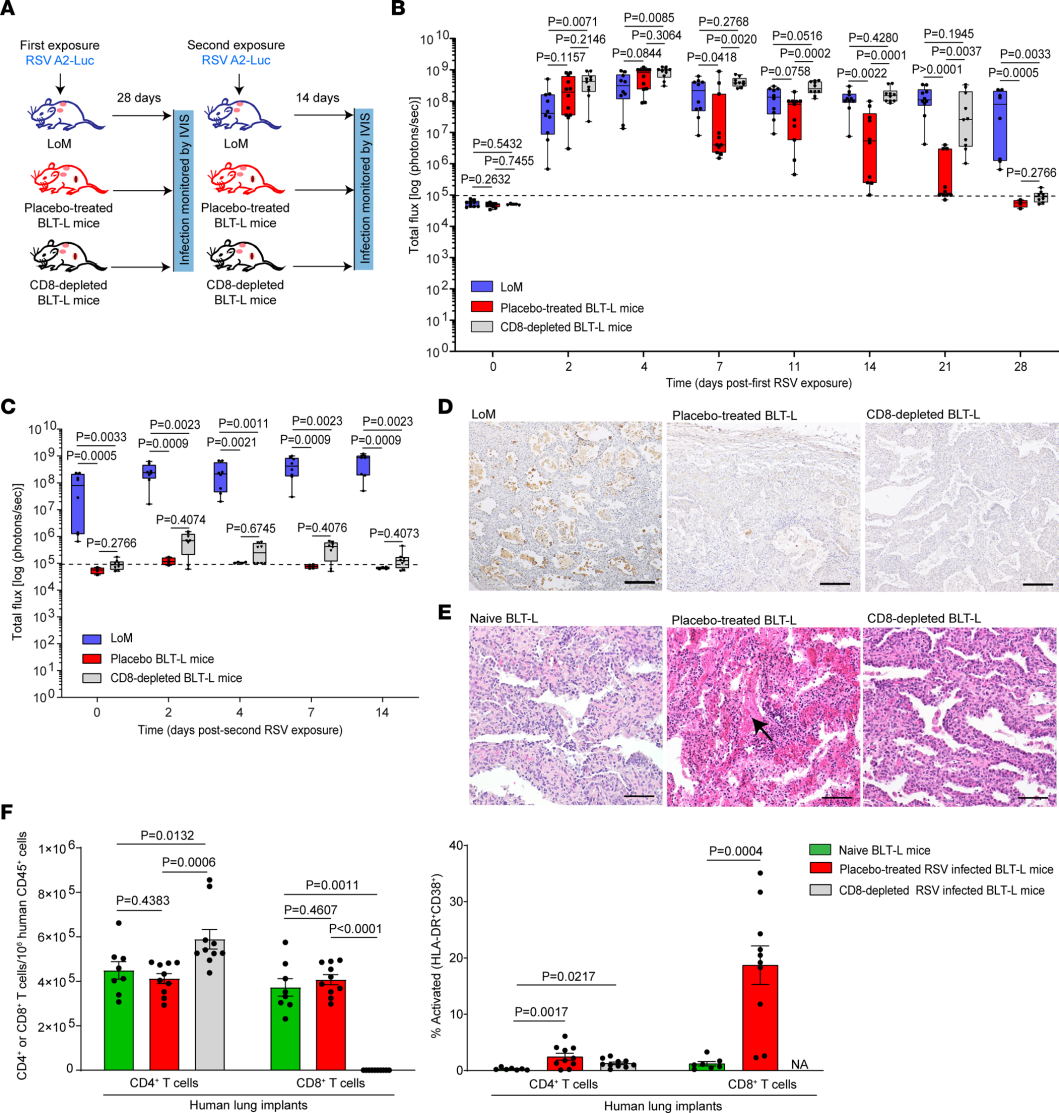
Human CD8^+^ T cell depletion impairs but does not eliminate the control of RSV replication in vivo. (**A**) Experimental diagram to evaluate the effect of CD8^+^ T cell depletion on RSV replication. LoM and BLT-L mice administered placebo or CD8-depleting antibody (3 mg/kg, i.v.) were exposed to RSV-Luc and, 21 days later, rechallenged with RSV-Luc. CD8^+^ T cell depletion was confirmed in blood and tissues (Supplemental Figure 3). (**B** and **C**) RSV replication was monitored in the lung implants of LoM (blue boxes, *n* = 10 implants), placebo-treated BLT-L mice (red boxes, *n* = 12 implants), and CD8-depleted BLT-L mice (gray boxes, *n* = 10 implants) following the (**B**) first and (**C**) second RSV exposure by measuring the bioluminescence signal (radiance [p sec^–1^ cm^–2^ sr^–1^] represented as total flux). The median (horizontal line), upper and lower quartiles (box ends), and minimum to maximum values (whiskers) are shown. (**D**) RSV antigen (brown) in the lung implants of LoM, placebo-treated BLT-L mice, and CD8-depleted BLT-L mice 14 days after the second exposure (*n* = 6 implants analyzed). Scale bars: 100 μm. (**E**) H&E staining of the lung implants of naive (*n* = 5 implants analyzed), placebo-treated (*n* = 4 implants analyzed), or CD8-depleted (*n* = 8 implants analyzed) BLT-L mice. Scale bars: 100 μm. Arrow indicates fibrin in airway lumen. (**F**) CD4^+^ and CD8^+^ T cell numbers (left) and activated (CD38^+^HLA-DR^+^) CD4^+^ and CD8^+^ T cell levels (right) in the lung implants of naive BLT-L mice (green; T cell levels, *n* = 8 implants; activation levels, *n* = 7 implants) and in placebo-treated (red, *n* = 10 implants) and CD8-depleted (gray, *n* = 10 implants) BLT-L mice 14 days after the second RSV exposure. Data are shown as mean ± SEM. Statistical significance was determined with a 2-tailed (**B**, **C**, and **E**) Kruskal-Wallis or (**E**) Mann-Whitney *U* test. *P* values were adjusted for multiple testing using the Benjamini, Krieger, and Yekutieli FDR method.

To evaluate the contribution of CD8^+^ T cells to the control of RSV infection in vivo, we first established the presence of RSV-specific, HLA-restricted CD8^+^ T cells in the human lung implants of RSV-infected BLT-L mice as determined by reactivity with RSV pentamer (Figure 3H). Next, BLT-L mice were administered a CD8^+^ T cell–depleting antibody or placebo twice weekly (27–29) (Figure 4A). CD8^+^ T cell depletion was confirmed in peripheral blood and tissues (Supplemental Figure 3). As a control, human donor–matched LoM were exposed to RSV A2-Luc (Figure 4A). RSV infection was readily detected at 2 days after inoculation in the lung implants of LoM and BLT-L mice. Peak levels of RSV infection were observed at 4 days after inoculation (LoM, 3.8 × 10^8^ ± 1 × 10^8^ [mean ± SEM] radiance; placebo-treated BLT-L mice, 6.5 × 10^8^ ± 1.2 × 10^8^ [mean ± SEM] radiance; CD8-depleted BLT-L mice, 8.43 × 10^8^ ± 9.8 × 10^7^ [mean ± SEM] radiance) (Figure 4B). In agreement with our earlier findings, RSV infection was sustained in LoM over the course of the experiment (Figure 4B). In RSV-infected BLT-L mice administered placebo (non–CD8-depleted), RSV infection was decreased by ~1 log by 11 days after inoculation, and RSV-Luc bioluminescence reached background levels by 28 days after inoculation (Figure 4B). RSV infection was also controlled in CD8-depleted BLT-L mice, albeit with a slight delay (Figure 4B). Specifically, no significant difference was observed in RSV infection levels between LoM and CD8-depleted BLT-L mice through 21 days after inoculation. In CD8-depleted BLT-L mice, RSV-Luc bioluminescence was ~2 logs higher at 21 days after inoculation (9 × 10^7^ ± 3.9 × 10^7^ [mean ± SEM] radiance; *P* = 0.0037) compared with BLT-L mice administered placebo (1.07 × 10^6^ ± 4.9 × 10^5^ [mean ± SEM] radiance). However, RSV-Luc bioluminescence in CD8-depleted BLT-L mice decreased to background levels by 28 days after inoculation (Figure 4B). Collectively, these results indicate that, while CD8^+^ T cells contribute to the control of primary RSV infection in BLT-L mice, they are not the sole mediators of virus clearance.

### CD8^+^ T cells offer protection against a second RSV challenge.

To evaluate the contribution of CD8^+^ T cells to immune-mediated protection against a second RSV challenge, CD8-depleted BLT-L mice, placebo-treated BLT-L mice, and LoM were rechallenged with RSV A2-Luc (Figure 4A). Following the second virus inoculation, the RSV-Luc bioluminescence signal in LoM increased approximately 3-fold by 2 days after inoculation and was sustained at high levels. In contrast, the RSV-Luc bioluminescence signal in placebo-treated BLT-L mice increased slightly just above background levels at 2 days after inoculation (1.5 × 10^5^ ± 1.8 × 10^4^ [mean ± SEM] radiance) but then quickly returned to background levels, indicating that RSV infection was rapidly controlled (Figure 4C). RSV infection was also detected in CD8-depleted BLT-L mice 2 days after the second virus challenge (Figure 4C), although RSV levels were ~3 logs lower (7.4 × 10^5^ ± 1.8 × 10^5^ [mean ± SEM] radiance) than that observed 2 days after the first virus challenge (4.4 × 10^8^ ± 9.3 × 10^7^ [mean ± SEM] radiance) (Figure 4, B and C). RSV levels then decreased over time, and RSV-Luc bioluminescence in CD8-depleted BLT-L mice reached background levels by 14 days after inoculation of the second virus challenge (Figure 4C). At necropsy (28 days after second RSV inoculation), few if any RSV-infected cells were detected in the human lung implants of CD8-depleted or placebo-treated BLT-L mice, consistent with the control of RSV infection following a second virus challenge (Figure 4D). RSV-infected cells were clearly present in the lung implants of LoM (Figure 4D). These results demonstrate that CD8^+^ T cells contribute to protection against a second homologous virus challenge. Interestingly, fibrin was observed in the airway lumen of the lung implants of placebo-treated BLT-L mice but not CD8-depleted BLT-L mice (or naive BLT-L mice) (Figure 4E), suggesting that human CD8^+^ T cells might also contribute to human lung pathology following RSV exposure.

### CD8^+^ T cells and CD4^+^ T cells clear RSV infection in vivo.

**Figure 5:**
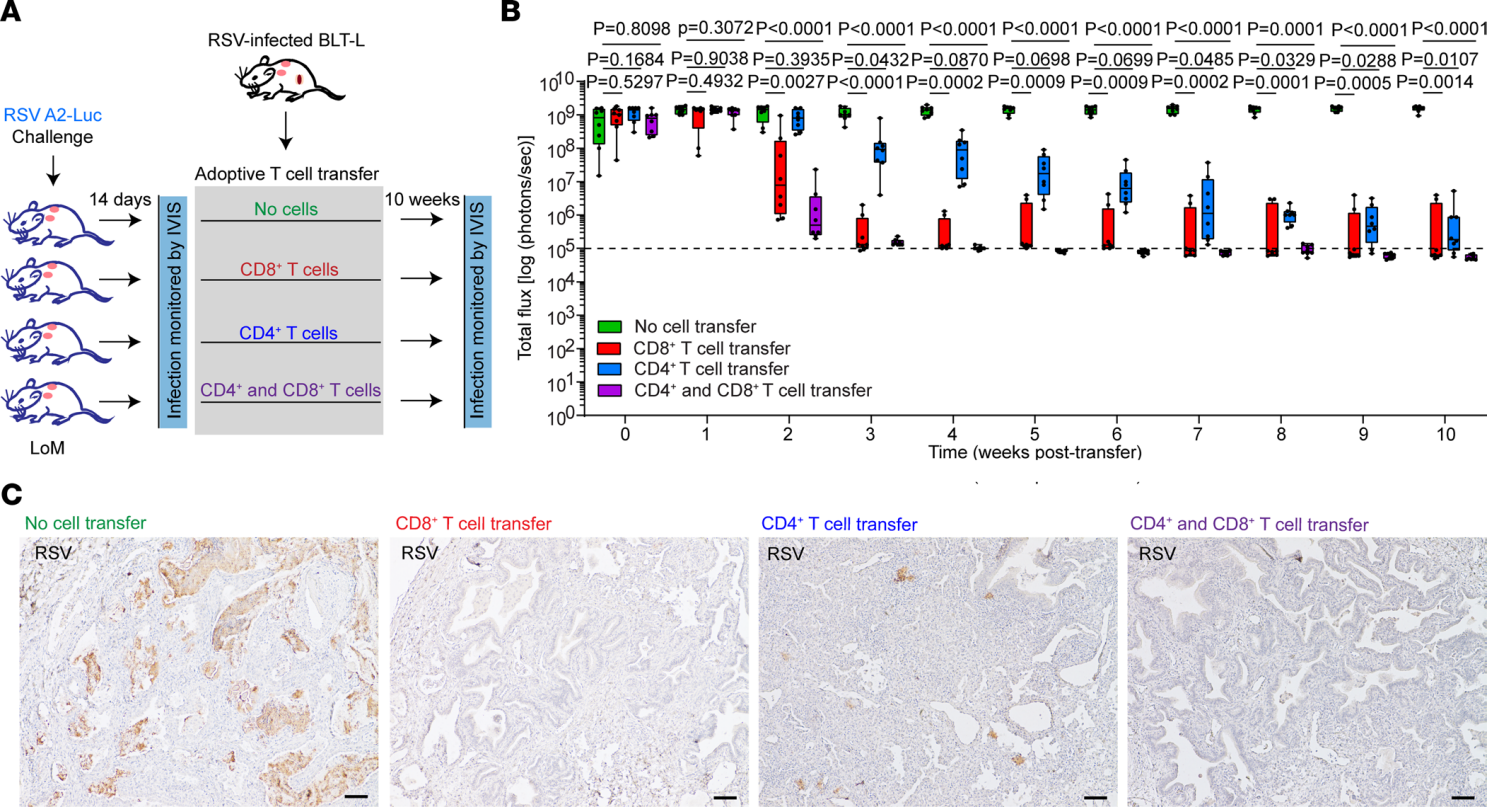
Adoptively transfered primed autologous human CD4^+^ or CD8^+^ T cells control RSV replication in vivo. (**A**) Experimental diagram to evaluate RSV replication in LoM following adoptive transfer of primed autologous CD4^+^ and CD8^+^ T cells. LoM were challenged with RSV-Luc and then, 14 days later, transplanted with autologous CD4^+^ T cells (15 × 10^6^), CD8^+^ T cells (15 × 10^6^), or both CD4^+^ (7.5 × 10^6^) and CD8^+^ (7.5 × 10^6^) T cells isolated from RSV-infected human donor-matched BLT-L mice. RSV-infected LoM that did not receive cells served as a control for RSV replication. (**B**) Longitudinal analysis of RSV replication in LoM that received no cells (green boxes, *n* = 8 implants), CD8^+^ T cells (red boxes, *n* = 8 implants), CD4^+^ T cells (blue boxes, *n* = 8 implants), or both CD4^+^ and CD8^+^ T cells (purple boxes, *n* = 8 implants) as measured by bioluminescence signal (radiance [p sec^–1^ cm^–2^ sr^–1^] represented as total flux). Dashed line indicates threshold for bioluminescence detection. The median (horizontal line), upper and lower quartiles (box ends), and minimum to maximum values (whiskers) are shown. Statistical significance was compared with a 2-tailed Kruskal-Wallis test. *P* values were adjusted for multiple testing using the Benjamini, Krieger, and Yekutieli FDR method. (**C**) RSV antigen staining (brown) in the human lung implants of RSV-infected LoM 10 weeks after adoptive transfer of no cells, CD8^+^ T cells, CD4^+^ T cells, or both CD4^+^ and CD8^+^ T cells (*n* = 4 implants analyzed/group). Scale bars: 100 μm.

In the absence of CD8^+^ T cells, infection is eventually cleared in BLT-L mice by other immune components. Further analysis of BLT-L mice challenged twice with RSV indicated that significantly higher numbers of CD4^+^ T cells were present in the human lung implants of CD8-depleted BLT-L mice (5.8 × 10^5^ ± 4.3 × 10^4^ [mean ± SEM]) compared with RSV-infected placebo-treated BLT-L mice (4.1 × 10^5^ ± 2.2 × 10^4^ [mean ± SEM], *P* = 0.0006) and donor-matched naive controls (4.4 × 10^5^ ± 3.9 × 10^4^ [mean ± SEM], *P* = 0.0132) (Figure 4F and Supplemental Figure 3D). The levels of activated CD4^+^ T cells in the lung implants of RSV-infected CD8-depleted mice (1.28% ± 0.23% [mean ± SEM], *P* = 0.0217) and placebo-treated BLT-L mice (2.46% ± 0.61% [mean ± SEM], *P* = 0.0017) were also higher compared with naive controls (0.28% ± 0.07% [mean ± SEM]) (Figure 4F). These results suggest that CD4^+^ T cells contributed to the control of RSV infection in CD8-depleted BLT-L mice. To directly determine the individual contribution of CD4^+^ T cells and CD8^+^ T cells to the control of RSV infection in vivo, LoM were inoculated with RSV A2-Luc and, 14 days later, administered purified CD4^+^ T cells, CD8^+^ T cells, or both CD4^+^ T cells and CD8^+^ T cells (CD4^+^/CD8^+^ T cells) purified from RSV-infected human donor–matched BLT-L mice (Figure 5A and Supplemental Figure 4, A and B). RSV infection in lung implants was monitored longitudinally by measuring RSV-Luc bioluminescence. RSV-infected LoM not administered cells served as a control (Figure 5A).

At 1 week after adoptive T cell transfer, equivalent levels of RSV infection were observed in the lung implants of nontransplanted controls and animals that received primed CD4^+^ T cells, CD8^+^ T cells, or both CD4^+^ and CD8^+^ T cells (*P* = 0.7093) (Figure 5B). However, at 2 weeks after T cell transfer, RSV infection decreased by 2.3 logs in LoM transplanted with CD4^+^/CD8^+^ T cells compared with nontransplanted controls (*P* < 0.0001) (Figure 5B). RSV levels continued to decline in CD4^+^/CD8^+^ T cell–transplanted LoM, and the RSV-Luc bioluminescence signal approached background levels by 4 weeks after T cell transfer (Figure 5B). No RSV antigen was readily detected by IHC in the lung implants of CD4^+^/CD8^+^ T cell–transplanted LoM analyzed at necropsy (10 weeks after T cell transfer) (Figure 5C). These results indicate that human T cells can effectively control RSV infection in vivo in the absence of an RSV-specific antibody response.

RSV infection was also significantly lower in CD8^+^ T cell–transplanted LoM at 2 weeks after T cell transfer compared with nontransplanted controls (*P* = 0.0027) (Figure 5B). RSV-Luc bioluminescence then decreased to near-background levels by 4 weeks after T cell transfer in the human lung implants of all but 1 CD8^+^ T cell transplanted LoM (Figure 5B). This LoM continued to have a dramatically reduced (~2.5 logs) but detectable bioluminescence signal in both lung implants, despite the continued presence of CD8^+^ T cells in the tissues, as demonstrated at necropsy (Figure 5B and Supplemental Figure 4, C and D). Clearance of RSV infection in the lung implants of CD8^+^ T cell–transplanted LoM with undetectable RSV-Luc bioluminescence at necropsy was confirmed with IHC by the absence of detectable RSV antigen (Figure 5C). These results indicate that primed CD8^+^ T cells effectively control RSV infection in vivo again, in the absence of a RSV-specific antibody response.

RSV levels in the human lung implants of CD4^+^ T cell–transplanted mice were not significantly different from nontransplanted controls at 2 weeks after T cell transfer (*P* = 0.3935) (Figure 5B). However, RSV-Luc bioluminescence was significantly reduced in CD4^+^ T cell–transplanted mice at 4 weeks after inoculation (*P* = 0.0870) but not to background levels (Figure 5B). A gradual reduction in RSV infection was observed in CD4^+^ T cell–transplanted mice. At 10 weeks after T cell transfer, the RSV-Luc bioluminescence signal in CD4^+^ T cell–transplant mice (9.6 × 10^5^ ± 6.3 × 10^5^ [mean ± SEM] radiance) was 3.2 logs lower than nontransplanted controls (1.5 × 10^9^ ± 1.0 × 10^8^ [mean ± SEM] radiance, *P* = 0.0107) (Figure 5B). While RSV-infected cells could still be detected in the lung implants of some CD4^+^ T cells transplanted LoM at necropsy, the abundance of RSV antigen in the human lung implants was dramatically lower than that observed in nontransplanted controls (Figure 5C).

The ability of CD4^+^ T cells to control RSV infection in vivo was confirmed in a separate experiment using half the number of adoptively transferred human CD4^+^ T cells isolated from either RSV-infected or uninfected animals (Supplemental Figure 5, A–E). Only CD4^+^ T cells isolated from RSV-infected animals were able to reduce RSV levels in LoM following adoptive transfer (Supplemental Figure 5C). The kinetics of RSV infection control in LoM that were transplanted with CD4^+^ T cells from RSV-infected BLT-L mice was similar to that observed in LoM transplanted with twice as many CD4^+^ T cells (Figure 5B), as measured by RSV-Luc bioluminescence. No reduction in RSV infection was observed in RSV-infected LoM following the adoptive transfer of CD4^+^ T cells isolated from naive BLT-L mice (Supplemental Figure 5, F–J). Despite the sustained presence of naive CD4^+^ T cells in human lung implants and all other tissues analyzed from LoM at 10 weeks after CD4^+^ T cell transfer, IHC revealed an abundance of RSV antigen in human lung implants (Supplemental Figure 5, I and J). These data indicate that primed CD4^+^ T cells can control RSV infection in vivo in the absence of CD8^+^ T cells, albeit with slower kinetics. Overall, based on our collective data, we propose that while CD8^+^ T cells or CD4^+^ T cells alone are sufficient to control RSV infection, both are required for more rapid clearance of virus infection in vivo.

## Discussion

An effective and safe RSV vaccine is a priority for the WHO Initiative for Vaccine Research, especially for infants and young children (17, 30). The incomplete understanding of how the human immune response controls RSV infection has proven to be a major hurdle toward developing an effective vaccine (31, 32). Three candidate RSV vaccines developed by Pfizer, Moderna, and GlaxoSmithKline have shown efficacy against RSV-associated lower respiratory tract disease in infants of vaccinated mothers and/or older individuals in phase III clinical trials. The US Food and Drug Administration (FDA) recently recommended approval of Pfizer and GlaxoSmithKline vaccines for older individuals. These vaccines are all primarily designed to elicit neutralizing antibodies. While these results are promising and a step forward, none of these vaccine candidates are 100% effective at preventing lower respiratory tract disease (approximately 66%–83% effective), suggesting that the neutralizing antibody levels generated are not always sufficient to prevent infection and disease (15, 33–35). It also remains to be determined if vaccine efficacy will fluctuate during subsequent RSV seasons due to variations in the circulating strains and how long protection will last. This has been an issue with vaccines to other respiratory viruses like SARS-CoV-2. There is also a concerning potential association between the Pfizer vaccine and Guillain-Barré syndrome (36). A vaccine-induced RSV-specific T cell response would not be able to prevent infection, but it could accelerate virus clearance and ameliorate disease if vaccine-elicited antibodies fail to prevent infection. Here, we used 2 potentially novel models of RSV infection to gain detailed insight into how the human immune system, and in particular human T cells, controls and clears RSV infection (18). Unlike LoM, which sustained RSV infection, BLT-L mice controlled and cleared RSV infection by virtue of having an incorporated functional human adaptive immune system. In humans, immunocompetent patients can effectively clear RSV infection and elicit a protective immune response, which limits RSV infection following a second challenge (37). Although RSV-specific IgM and IgG were detected in BLT-L mice — consistent with previous studies indicating that, in humans, the humoral immune system contributes to the overall response to RSV infection (37–39) — the complete control of RSV infection in LoM transplanted with RSV-specific CD4^+^ and CD8^+^ T cells highlights the significance of an effective T cell response on controlling and eliminating RSV infection.

Suitable models that recapitulate RSV infection of the human respiratory tract for the development of antiviral and vaccine approaches are highly desired. The human RSV challenge model in which the nasal epithelium of human volunteers is inoculated with RSV under closely monitored conditions can provide unique insight into RSV infection and disease outcomes (40). However, this approach is expensive, requires specialized facilities and staff, and requires that more invasive sampling (e.g., tissue biopsies) must be kept to a minimum (40). In addition, experiments involving immune cell depletion/transplantation to directly examine the role of specific immune components in RSV infection are not ethical to perform in humans. Multiple in vitro models utilizing human epithelial cells have been used to investigate RSV infection kinetics and identify strategies to reduce virus infection, including human-derived cell lines and 3D planar or organoid models of human airway epithelium (41–45). However, these culture systems lack components of the immune system important during RSV infection, such as macrophages, neutrophils, DCs, and lymphocytes (42). Although more sophisticated culture models are being developed that incorporate some of these immune cell populations, the successful demonstration of epithelial cell–immune cell interactions is limited. Precision cut lung slices (PCLS) provide the regional distribution of differentiated epithelial cell types important for RSV infection (46, 47). Since PCLS also contain nonepithelial lung cell populations such as tissue resident immune cells, smooth muscle cells, and vascular endothelial cells, these models have the potential to assess RSV infection beyond epithelial cells. The usefulness of PCLS depends on the availability of suitable human lung tissue and the optimization of the conditions to maintain the tissue in culture (up to 14 days) (47). PCLS also lack systemic immune cells normally recruited into the lung during RSV infection.

The human lung implants in LoM and BLT-L mice contain a broad range of human lung cells including airway epithelial cells, alveolar epithelial cells, smooth muscle cells, and vascular endothelial cells for the in vivo study of viral and bacterial infection, including RSV (18, 19). LoM and BLT-L mice provide a unique system to study RSV pathogenesis in the bronchiolar airways and alveolar lung regions and this pathogenesis is responsible for the more severe RSV infections especially in infants (21). In other studies with HCMV, we previously demonstrated that the lung implants of LoM can sustain virus replication over time, while virus replication is controlled in BLT-L mice due to the presence of an autologous systemic human immune system capable of eliciting antigen-specific antibody and T cell responses (18). Therefore, LoM provide a preclinical system to study virus infection in the absence of full adaptive immunity and in combination with BLT-L mice, immune correlates of efficient virus clearance. While our data demonstrating sustained RSV replication in LoM indicate that the innate immune response is not sufficient to control RSV infection in vivo, whether or not innate immune cells like neutrophils contribute to virus clearance by modulating the adaptive immune response needs to be examined. A limitation of LoM and BLT-L mice for the study of RSV infection is that the human lung implants are an enclosed organ system without mucociliary or cough clearance mechanisms. These mechanisms facilitate the clearance of cellular debris and mucous secretions. However, despite lacking this important innate defense mechanism, we demonstrated that RSV infection is effectively cleared in BLT-L mice, thus highlighting the importance of adaptive immunity for elimination of RSV infection. While nonadult tissues are used to construct LoM and BLT-L mice, they are transplanted into adult human mice. Although these models recapitulate features of RSV infection in human infants, such as the production of proinflammatory cytokines and the recruitment and accumulation of neutrophils into airway lumen, it is currently not known if the use of adult mice will affect the ability of the models to recapitulate other aspects of infant RSV infection.

Patients with the highest neutralizing antibody levels are not always protected from subsequent RSV infection, and T cells have been shown to be essential for clearance of intracellular pathogens and the development of long-lived adaptive immune responses (48–52). In fact, children with primary deficiencies affecting T cell function suffer more severe and prolonged RSV infections (32, 37–39). Our results demonstrating that human T cells can clear RSV infection in vivo are also congruent with studies performed in immunocompetent mouse models of RSV infection, where RSV-infected mice were administered RSV-specific CD4^+^ T cell and/or CD8^+^ T cell lines (53–56). However, studies in immunocompetent mouse models suggest that RSV-specific T cells might promote disease and induce lung pathology, including features of early diffuse alveolar damage (e.g., alveolar hemorrhage, hyaline membrane formation, and necrosis) (53–56). The adoptive transfer of RSV-specific mouse CD8^+^ T cells into nude mice was shown to mediate lethal lung pathology (56). In sharp and important contrast, T cells have not been shown to mediate lung pathology in human RSV infections. Specifically, examination of RSV-specific T cells during experimental RSV infection of adults failed to demonstrate an association with RSV-specific CD8^+^ T cell frequencies and immunopathology (57). In our studies, we demonstrate that RSV infection in BLT-L mice induced an RSV-specific CD8^+^ T cell response to virus nucleoprotein as determined by pentamer staining. The limited availability of pentamers specific for HLA-RSV peptide complexes prevented us from further exploring the CD8^+^ T cell response against other RSV immunodominant epitopes from RSV matrix protein (M), F protein, and glycoprotein (G). Nevertheless, we demonstrated that adoptive transfer of primed human CD8^+^ T cells effectively controlled RSV replication in LoM, further confirming that primed CD8^+^ T cells are important effector cells in controlling RSV infection. Consistent with the minimal contribution of the mouse thymus to human T cell production in BLT mice compared with the human thymic organoid (58), the vast majority of T cells present in this model are produced in the human thymic organ and are HLA restricted. Interestingly, in sharp contrast to some studies in mice, neither the adoptive transfer of antigen-experienced human T cells into LoM nor the reexposure of BLT-L mice to RSV induced lethal pathology. While we did not readily observe the presence of hyaline membranes, we noted fibrin deposition in the airway spaces of BLT-L mice challenged twice with RSV but not when mice were administered CD8-depleting antibody. These results indicate that human CD8^+^ T cells do not mediate overt human lung pathology during RSV infection of human lung implants — at least not to the levels observed in mouse models (53–56). It is therefore possible that, during RSV infection of healthy individuals, CD8^+^ T cell–mediated lung pathology is minimal and quickly resolved.

There is a growing body of data suggesting that CD4^+^ T cells also contribute to RSV clearance in animal models, with CD4^+^ T cell depletion resulting in prolonged viral shedding (39, 54). The control of RSV infection in CD8^+^ T cell–depleted BLT-L mice and higher frequencies of activated CD4^+^ T cells in human lung implants led us to hypothesize that CD4^+^ T cells could directly contribute to RSV clearance in addition to enhancing antibody-mediated immunity. To test our hypothesis, we purified CD4^+^ T cells from primed RSV-infected BLT-L mice and performed an adoptive transfer into RSV-infected donor–matched LoM. We observed that RSV-primed CD4^+^ T cells but not RSV-naive CD4^+^ T cells were able to control RSV replication in vivo. Since the CD4^+^ T cell–depleting antibodies available are not broadly effective (59, 60), based on our CD8^+^ T cell depletion and adoptive T cell transfer experiments, we predict that CD4^+^ T cell depletion of BLT-L mice prior to RSV exposure would delay the control of RSV infection due to their enhancement of B cell–mediated immunity and direct effect on RSV-infected cells.

In summary, our results demonstrate that primary RSV infection can elicit a protective human immune response that confers protection from a second RSV challenge. RSV vaccines focused on neutralizing antibody production are challenged by the natural diversity that exists among circulating RSV strains in the major antigenic sites of the commonly targeted RSV F protein. The demonstration that CD4^+^ and CD8^+^ T cells have critical yet independent roles in controlling RSV infection in the absence of an antibody response suggests that vaccines that also elicit CD4^+^ and CD8^+^ T cell immunity may provide long-term protection against RSV infection and limit the severity of subsequent lung disease.

## Methods

### Experimental design.

RSV infection was analyzed in LoM and BLT-L mice following injection of virus expressing GFP or luciferase reporter genes into human lung implants. The human lung implants of mice were analyzed at predetermined time points by in vivo imaging (luciferase activity), histology, real-time PCR, flow cytometry, and/or titer to assess virus infection, pathogenesis, and immune cells. RSV-specific immune cell responses were measured in RSV-infected BLT-L mice via ELISA (antibody response) or tetramer staining and flow cytometry (T cell response). Human T cell depletion and adoptive T cell transfer studies were performed to analyze the role of human T cells in RSV clearance in vivo.

### Generation of humanized mice.

LoM and BLT-L mice (Supplemental Table 1) were generated as described previously (18, 19). In brief, LoM were constructed by implanting 2 pieces of human lung tissue (Advanced Bioscience Resources) s.c. into the back of 8- to 17-week-old male and female NOD.Cg-Prkdcscid ll2rgtm1Wjl/SzJ mice (NSG mice; The Jackson Laboratory). After 10 weeks, LoM were used for experimentation. This allows time for the incision site on the animal to heal and the implanted lung tissue to vascularize and to expand so that it is readily palpable and a suitable size for injection. To construct BLT-L mice, 10- to 15-week-old male and female NSG mice were first irradiated (200 rad), and then human thymus-liver-thymus tissue (Advanced Bioscience Resources) was implanted under the kidney capsule. Two pieces of autologous human lung tissue were implanted s.c. into the right and left back. Following tissue implantation, animals were transplanted with autologous liver–derived human CD34^+^ hematopoietic stem cells (via tail vein injection). The non–CD34^+^ cell fraction was used for 4-digit HLA typing at the HLA-A and B loci. Reconstitution of BLT-L mice with human hematopoietic cells was monitored longitudinally by flow cytometry as previously described (18). Mice were maintained by the Division of Comparative Medicine at UNC Chapel Hill under specific pathogen–free conditions.

### Viruses and in vivo analysis of infection.

Recombinant RSV A2-GFP (61) or RSV A2-Luc reporter genes inserted between the RSV phosphoprotein (*P*) and matrix protein (*M*) genes were obtained from Viratree and were directly inoculated into the human lung implants of anesthetized LoM and BLT-L mice (2.5 × 10^5^ TCID_50_ per human lung implant, 100 μL volume). At necropsy, human lung implants were collected, enzymatically digested, and passed through a cell strainer as described previously (18). RSV infection and replication following exposure to RSV-A2 GFP were evaluated by measuring GFP expressing cells with flow cytometry. Data were collected on a BD LSRFortessa instrument (BD Biosciences) and analyzed with BD FACSDiva (version 6.1.3, BD Bioscience) and FlowJo (version 10.6.2, FlowJo LLC) software. Live imaging of RSV A2-Luc–infected mice was performed using an in vivo imaging system (IVIS).

### qPCR and viral titer determination for RSV.

Human lung implants were cut into small pieces and then further homogenized using a Minilys Tissue Homogenizer (Bertin Instruments). Tissues were ground at medium speed twice for 30 seconds, once at high speed for 45 seconds, and then twice at high speed for 30 seconds. Each sample was then divided into two 2 mL reinforced tubes prepared with zirconium oxide beads and ground in a MiniLys Tissue Homogenizer at medium speed twice for 30 seconds. Samples were then centrifuged at 240 x g for 5 minutes at 4°C. In total, 200 μL of homogenate supernatant was then combined with 600 μL Trizol (Ambion by Life Technologies). Samples were vortexed thoroughly and RNA extracted using the Direct-zol RNA Miniprep kit (Zymo Research) according to the manufacturer’s protocol. RNA concentrations were measured using a NanoDrop One (Thermo Fisher Scientific). This concentration was used to calculate the volume of solution containing 500 ng RNA for each sample. This volume was then used to generate cDNA using iScript Reverse Transcription Supermix for quantitative PCR (qPCR) (Bio-Rad). The resulting cDNA was diluted 20-fold in Tris-EDTA buffer, pH 8.0 (Amresco). qPCR was performed using SsoAdvanced Universal Probes Supermix or SsoAdvanced Universal SYBR Green Supermix (depending on the primers used) according to the manufacturer’s protocol (Bio-Rad). Relative quantification was performed using Quant6 Studio Flex software based on the crossing point (Cp) value that defines the cycle number at which the fluorescence signal of the sample exceeds a background fluorescence value. A lower Cp correlated with higher target expression. To determine RSV titers in human lung implants, HEp-2 cells (ATCC) were seeded onto 96-well plates at a density of 20,000 cells/well and cultured with MEM (Gibco) supplemented with 10% FBS (Avantor) and 1% pen/strep (Sigma). Media were aspirated from each well after 24 hours and replenished with 180 μL of media. Ten-fold serial dilutions of lung homogenate were then added to wells (20 μL/well), and titers were determined 5–7 days later. RSV viral titers were determined using a TCID_50_ assay on HEp-2 cell (ATCC) monolayers, with positive wells scored by GFP expression.

### In vivo luminescence measurements.

Imaging was performed with a cooled CCD camera (Xenogen IVIS-Lumina, Perkin Elmer). D-Luciferin (Perkin Elmer) (15 mg/kg, i.p. injection) was administered 5 minutes prior to imaging. Mice were placed in an induction chamber and anesthetized with isoflurane (2% in oxygen). Background luminescence in human lung implants was determined in LoM and BLT-L mice before RSV exposure. Bioluminescence images were acquired for 3 minutes with f/sto*P* (amount of light collected via aperture opening) = 1.2 and binning (grouping of pixels into a single larger pixel) = 4. Pseudo-colored images of photon emissions were overlaid on grayscale images of the whole mouse in order to assist with spatial localization of the luciferase signals. A digital false-color photon emission image of the mouse was generated, and photons were counted within a constant region of interest (ROI) corresponding to the human lung implant. Photon emission was measured as radiance in p s^−1^ cm^−2^ sr^−1^ and represented as total flux (photons/sec).

### Flow cytometric analysis of immune cells.

Peripheral blood and tissues were collected from mice. Tissues were processed into single-cell suspensions as previously published (18). Human lung implants and mouse lungs were enzymatically digested prior to passing tissue through a cell strainer, RBCs were lysed, and then cells were counted and aliquoted for flow cytometry. Prior to antibody incubation, Ig-binding sites were blocked. The following antibody panel was used to analyze the mouse (m) immune cell subsets: mCD11b-APC-Cy7 (BD Biosciences, 557657, clone M1/70), mCD11c-BV786 (BD Biosciences, 563735, clone HL3), mCD24-BV711 (BD Biosciences, 563450, clone M1/69), mCD45-BV605 (BioLegend, 103139, clone 30-F11), mCD64-BV421 (BioLegend, 139309, clone X54-5), IA/IE-BV650 (BD Biosciences, 563415, clone M5/114.15.2), mLy6C-PerCP-Cy5.5 (Thermo Fisher Scientific, 45-5932-82, clone HK1), and mLy6G-Alexa Flour700 (BD Biosciences, 561236, clone 1A8). The antibody panels to analyze human (h) immune cells were as follows: hCD45-APC (BD Biosciences, 555485, clone HI30), hCD19-PE-Cy7 (BD Biosciences, 557835, clone SJ25C1), hCD33-PE (BD Biosciences, 347787, clone P67-6), hCD3-FITC (BD Biosciences, 555339, clone HIT3A), hCD4-APC-H7 (BD Biosciences, 560158, clone RPA-T4), and hCD8-PerCP (BD Biosciences, 347314, clone SK1) (flow cytometry antibody panel 1); and hCD45-V500 (BD Biosciences, 560777, clone HI30), hCD19-PE-Cy7 (BD Biosciences, 557835, clone SJ25C1), hCD3-AlexaFlour700 (Bio-Rad, MCA463A700, clone UCHT1) or APC-R700 (BD Biosciences, 565119, clone UCHT1), hCD4-APC-H7 (BD Biosciences, 560158, clone RPA-T4), hCD8-FITC (BD Biosciences, 340692, clone SK1), hCD45RA-Pacific Blue (Bio-Rad, MCA88PB, clone F8-11-13) or negative control mouse IgG1ak-Pacific Blue (BD Biosciences, 558120, clone MOPC-21), CD27-PE (BD Biosciences, 555441, clone M/T271) or negative control mouse IgG1k-PE (BD Biosciences, 555749, clone MOPC-21), HLA-DR-PerCP (BD Biosciences, 347364, clone L243) or negative control mouse IgG2ak-PerCP (BD Biosciences, 349054, clone X39), and CD38-APC (BD Biosciences, 340439, clone HB7) or negative control mouse IgG1k-APC (BD Biosciences, 555751, clone MOPC-21) (flow cytometry antibody panel 2). Following antibody incubation, PB was treated with 1***×*** BD FACS lysing solution (BD Biosciences) to remove red blood cells. Samples were then washed and fixed with PFA. Data were acquired on a BD LSRFortessa or FACSCanto instrument (BD Biosciences) and analyzed with FACSDiva software (version 6.1.3, BD Biosciences) and FlowJo (version 10.6.2, FlowJo LLC). The gating strategy to characterize the mouse immune cells was adapted from the previous published studies (62–64).

### Detection of IgM and IgG antibodies to RSV.

Antibodies to RSV in plasma were measured using an RSV IgM (Abcam, ab108766) or IgG (Abcam, ab108765) ELISA carried out as per manufacturer’s protocol. Standard units were calculated by the following formula: experimental (mean) absorbance value × 10/cut-off value. Samples are considered to give a positive signal if the standard units are > 11 (10% over the cut-off value) per the manufacturer’s protocol.

### Multimer reactivity.

HLA-A*02:01 PE–conjugated pentamer for RSV (KMLKEMGEV) (F801-2A-50 test R-phycoerythrin, ProImmune) was used for staining of total of 1 × 10^6^ cells from human lung implant suspensions. Cells were treated for 10 minutes with Human BD Fc Block (BD Biosciences) to prevent nonspecific staining. To separate viable cells, samples were stained with Zombie APC-Cy7 viability dye at room temperature for 20 minutes. Cells were then incubated for 15 minutes on ice with either HLA-A*0201/ KMLKEMGEV pentamer or negative control pentamer (HLA-matched EBV pentamer, ProImmune). Cells were further stained with the following antibody panel: hCD3-Pacific Blue (BD Biosciences, 558117, clone UCHT1), hCD4-BV605 (BD Biosciences, 562658, clone RPA-T4), hCD8-FITC (BD Biosciences, 340692, clone SK1), and PerCP-conjugated hCD14 (BioLegend, 325632, clone HCD14), hCD16 (BioLegend, 302030, clone 3G8), hCD19 (Biogend, 302228, clone HIB19), and hCD56 (BioLegend, 318342, clone HCD56) (dump channel) for 30 minutes at 4°C. Samples were then washed and fixed with PFA. Data were acquired on a BD LSRFortessa instrument (BD Biosciences) and analyzed with FACSDiva (version 6.1.3, BD Biosciences) or FlowJo (version 10.6.2, FlowJo LLC) software.

### T cell isolation from BLT-L mice for adoptive transfer.

Mononuclear cells (MNCs) were isolated from the BLT mouse spleen and liver as previously described (18) for enrichment of human CD4^+^ and CD8^+^ T cells. Each tissue was processed individually, and then tissue cells were pooled for immunomagnetic sorting. Each pooled tissue was first enriched for human cells with the EasySep Mouse/Human Chimera Kit (Stem Cell Technologies). First, CD4^+^ T cells were isolated using a human CD4^+^ selection kit (Miltenyi Biotec, 130-096-533). The unenriched cell population was then used to purify CD8^+^ T cells using a CD8^+^ T cell positive selection kit (Miltenyi Biotec, 130-045-20). Flow cytometry was performed before and after selection to confirm the efficacy of the sort and purity of the sorted samples.

### Adoptive T cell transfer.

LoM were infected with RSV-Luc A2 virus (2.5 × 10^5^ TCID_50_ per human lung implant) by intralung exposure 14 days prior to the adoptive T cell transfer. Human CD4^+^ T cells (15 × 10^6^ cells), CD8^+^ T cells (15 × 10^6^ cells), or both CD4^+^ T cells (7.5 × 10^6^) and CD8^+^ T cells (7.5 × 10^6^ cells) isolated from RSV-infected BLT mice were injected i.v. into human donor-matched RSV-infected LoM. LoM receiving an adoptive transfer of CD4^+^ T cells were treated every week with a CD8-depleting antibody (clone MT807R1, 3 mg/kg, i.v.) to deplete any residual human CD8^+^ T cells or NK cells (27). LoM receiving an adoptive transfer of human CD8^+^ T cells were treated every week with a CD4-depleting antibody (clone CD4R1, 6 mg/kg, i.v.) to deplete any residual human CD4^+^ T cells (65). In a separate experiment, RSV-infected LoM received an adoptive transfer of 7 ***×*** 10^6^ human CD4^+^ T cells isolated from human donor-matched naive BLT-L mice or twice-challenged RSV-infected BLT-L mice. RSV replication was monitored weekly by IVIS. After 10 weeks after adoptive transfer, mice were necropsied and human lung implants were collected for IHC and flow cytometric analysis. For flow cytometric analysis, MNCs were isolated from human lung implants following enzymatic digestion and passage over a cell strainer. Prior to antibody incubation Ig binding sites were blocked. The following antibody panel was used to analyze human immune T cell populations: hCD45-V500 (BD Biosciences, 560777, clone HI30), hCD19-PE-Cy7 (BD Biosciences, 557835, clone SJ25C1), hCD3-Alexa Flour 700 (Bio-Rad, MCA463A700, clone UCHT1) or APC-R700 (BD Biosciences, 565119, clone UCHT1), hCD4-APC-H7 (BD Biosciences, 560158, clone RPA-T4), hCD8-FITC (BD Biosciences, 340692, clone SK1), hCD45RA-Pacific Blue (Bio-Rad, MCA88PB, clone F8-11-13) or negative control mouse IgG1ak-Pacific Blue (BD Biosciences, 558120, clone MOPC-21), CD27-PE (BD Biosciences, 555441, clone M/T271) or negative control mouse IgG1k-PE (BD Biosciences, 555749, clone MOPC-21), HLA-DR-PerCP (BD Biosciences, 347364, clone L243) or negative control mouse IgG2ak-PerCP (BD Biosciences, 349054, clone X39), and CD38-APC (BD Biosciences, 340439, clone HB7) or negative control mouse IgG1k-APC (BD Biosciences, 555751, clone MOPC-21). Following antibody incubation, peripheral blood was treated with 1***×*** BD FACS lysing solution (BD Biosciences) to remove RBCs. Samples were then washed and fixed with PFA. Data were acquired on a BD LSRFortessa or FACSCanto instrument (BD Biosciences) and analyzed with FACSDiva (version 6.1.3, BD Biosciences) and FlowJo (version 10.6.2, FlowJo LLC) software.

### Histological staining.

Tissue samples obtained from LoM and BLT-L mice were fixed in 4% PFA or 10% formalin, paraffin embedded, and cut into 5 μm sections as previously described (18, 19). H&E staining was performed on tissue sections deparaffinized with xylene and graded ethanol. For IHC staining, prior to primary antibody incubation, antigen retrieval was performed, and Ig binding sites and endogenous peroxidase activity were blocked. Tissue sections were stained overnight at 4°C with primary antibodies directed against human hematopoietic cells (CD45, Agilent, M070101, clones 2B11 + PD7/26), T cells (CD3, Thermo Fisher Scientific, MA1-90581, clone SP7), CD4^+^ T cells (CD4, Genway Biotech, GWB-B38C3A, clone SP35), CD8^+^ T cells (CD8, Biocare, CRM311, clone SP35), and appropriate isotype-negative control antibodies (Agilent, X093101-2, clone DAK-G01; Agilent, X093602, clone rabbit immunoglobin). Tissue sections were developed using the MACH-3 polymer system (BioCare Medical) and 3,3′-diaminobenzidine (DAB) (Vector Laboratories). Polyclonal goat antibody directed against all RSV proteins was used to detect RSV antigen (Abcam, Ab20745). Tissue sections were developed using the ImmPRESS HRP Horse Anti-Goat IgG peroxidase Polymer Detection Kit (Vector Laboratories) and DAB. Subsequently, tissue sections were counterstained with hematoxylin and mounted. Tissue sections were imaged on a Nikon Eclipse Ci microscope using Nikon Elements BR software (version 4.30.01) with a Nikon Digital Sight DS-Fi2 camera or on an Olympus VS120 virtual slide scanning system and an Allied Vision Pike 5 CCD progressive scan camera using OlyVia software (version 2.9). Brightness, contrast, and white balance were adjusted on whole images using Adobe Photoshop (CS6).

### Statistics.

Sample sizes are indicated in the figure legends and Supplemental Table 1. No statistical methods were used to predetermine sample size. No inclusion/exclusion criteria were used. No data points were excluded from the analysis. No randomization was used to determine allocation of mice to experimental groups and samples to downstream analysis. The investigators were not blinded to group allocation for data collection or analysis. To ensure the appropriate reagents were used, information regarding the HLA type and infecting RSV strain were utilized. The human donor was controlled for BLT-L primary/secondary challenge experiments and for adoptive T cell transfer experiments. No other cofounders were controlled. Statistical parameters and tests are indicated in figure legends. Statistical tests were performed in GraphPad Prism (version 6). No methods were used to assess if data were parametric. Nonparametric tests were used due to the sample sizes. *P* values were adjusted for multiple testing, as indicated in figure legends. For all statistical comparisons, *P* < 0.05 was considered significant.

### Study approval.

Animal studies were carried out according to protocols approved by the IACUC at UNC Chapel Hill and in adherence to the *Guide for the Care and Use of Laboratory Animals* (National Academies Press, 2011).

### Data availability.

The data generated are available from corresponding authors upon reasonable request.

## Author contributions

CD inoculated mice with RSV; collected and processed samples from RSV-exposed mice; administered depleting antibodies to mice; analyzed samples collected from naive and RSV infected mice by flow cytometry and IHC; performed IVIS imaging; performed multimer staining; isolated cells for adoptive transfer experiments; performed RSV-specific antibody ELISAs and neutralization assays; conceived and designed experiments; and contributed to data interpretation, data presentation, and the preparation of the manuscript and its revision. RJP provided stocks of RSV A2-GFP and RSV-Luc; performed an analysis of human lung implant structures; conceived and designed experiments; and contributed to data interpretation, data presentation, and manuscript writing. CD and RJP are co–first authors based on their degrees of contribution. WY assisted with the inoculation of mice with RSV, performed IVIS imaging, collected and processed samples from mice, performed IHC staining for human immune cells and RSV antigen, and contributed to data interpretation. BL collected and processed samples from naive and RSV-exposed mice. AB processed and cultured tissue homogenate to determine the RSV titer, performed the RSV real-time PCR analyses, and analyzed data. MC assisted with the RSV-specific antibody ELISAs and neutralization assay. DMB performed H&E staining. GS assisted with CD8- and CD4-depletion studies. FBA contributed to the histological analysis. JKW contributed to data interpretation, data presentation, and manuscript writing. AW and JVG conceived and designed experiments and contributed to data interpretation, data presentation, and manuscript writing and coordinated the study, the preparation of the manuscript, and its revision.

## Supplementary Material

Supplemental data

Supplemental table 1

Supporting data values

## Version 1 05/09/2023

In-Press Preview

## Version 2 06/08/2023

Electronic publication
