# Supplementary material for: Human T cells efficiently control RSV infection
Source: JCI Insight. 2023 Jun 8;8(11):e168110. doi: 10.1172/jci.insight.168110 (PMC10393221; doi:10.1172/jci.insight.168110)
Supplement: Supplemental data [file jciinsight-8-168110-s153.pdf]

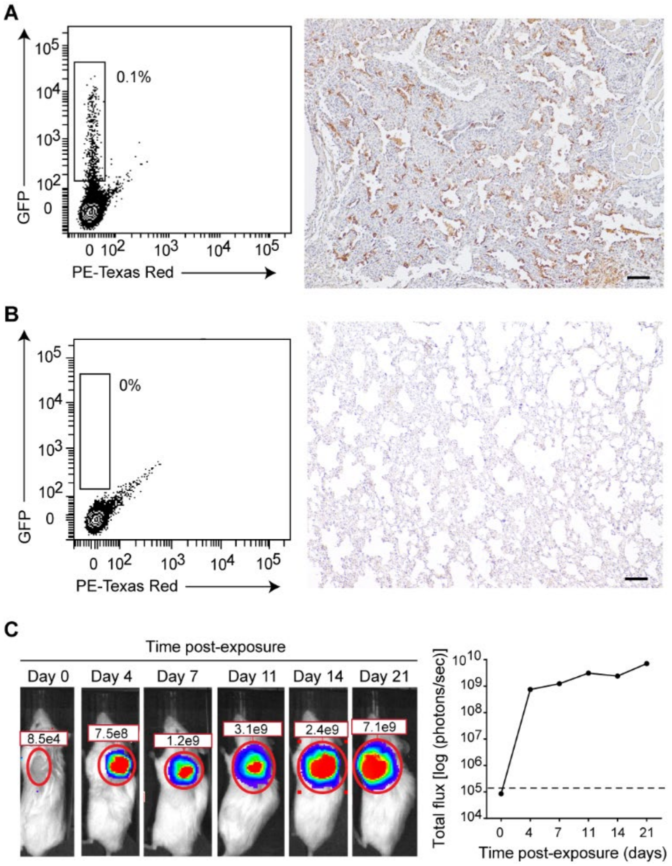

Supplemental Figure 1. RSV infection in LoM is restricted to human lung implants. LoM were infected with RSV via intra-lung exposure with recombinant RSV-A2-expressing GFP (2.5×10^5 $TCID_{50}$ per lung implant) and the (A) human lung implants and (B) mouse lungs analyzed (n=12 implants and 4 mouse lungs analyzed) for RSV infection at 4 days post-exposure by determining the number of GFP^+ cells with flow cytometry (left panels) and RSV antigen staining of tissue sections (right panels; positive cells, brown; scale bars, 100 um). Scale bars, 50 uM. (C) Representative IVIS imaging of a LoM infected with recombinant RSV-A2-expressing luciferase (left panel). Shown is the RSV luciferase bioluminescence signal (radiance [p sec^{-1} cm^{-2} sr^{-1}] represented as total flux) in the human lung implant prior to (day 0) and post RSV-exposure. Dashed line, threshold for bioluminescence detection (right panel).

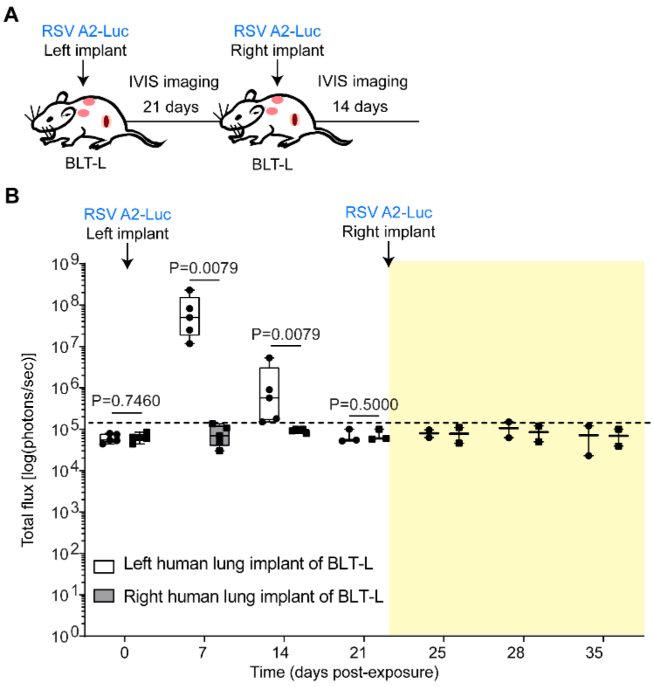

Supplemental Figure 2. Primary RSV infection elicits a protective systemic immune response

(**A**) The left human lung implant of BLT-L mice was inoculated with RSV-Luc and then 21 days later the right human lung implant of the same mice was inoculated with RSV-luc. (**B**) RSV replication was monitored longitudinally in the left (white bars, n=5) and right (gray bars, n=5) human lung implants of BLT-L mice by measuring the bioluminescence signal (radiance [p sec^{-1} cm^{-2} sr^{-1}] represented as total flux). Background luminescence measured pre-exposure is denoted by the dashed line. Yellow shading indicates the time corresponding to post-inoculation of the right human lung implant with RSV-luc. The median (horizontal line), upper and lower quartiles (box ends), and minimum to maximum values (whiskers) are shown. Statistical significance was determined with a two-tailed Mann–Whitney test.

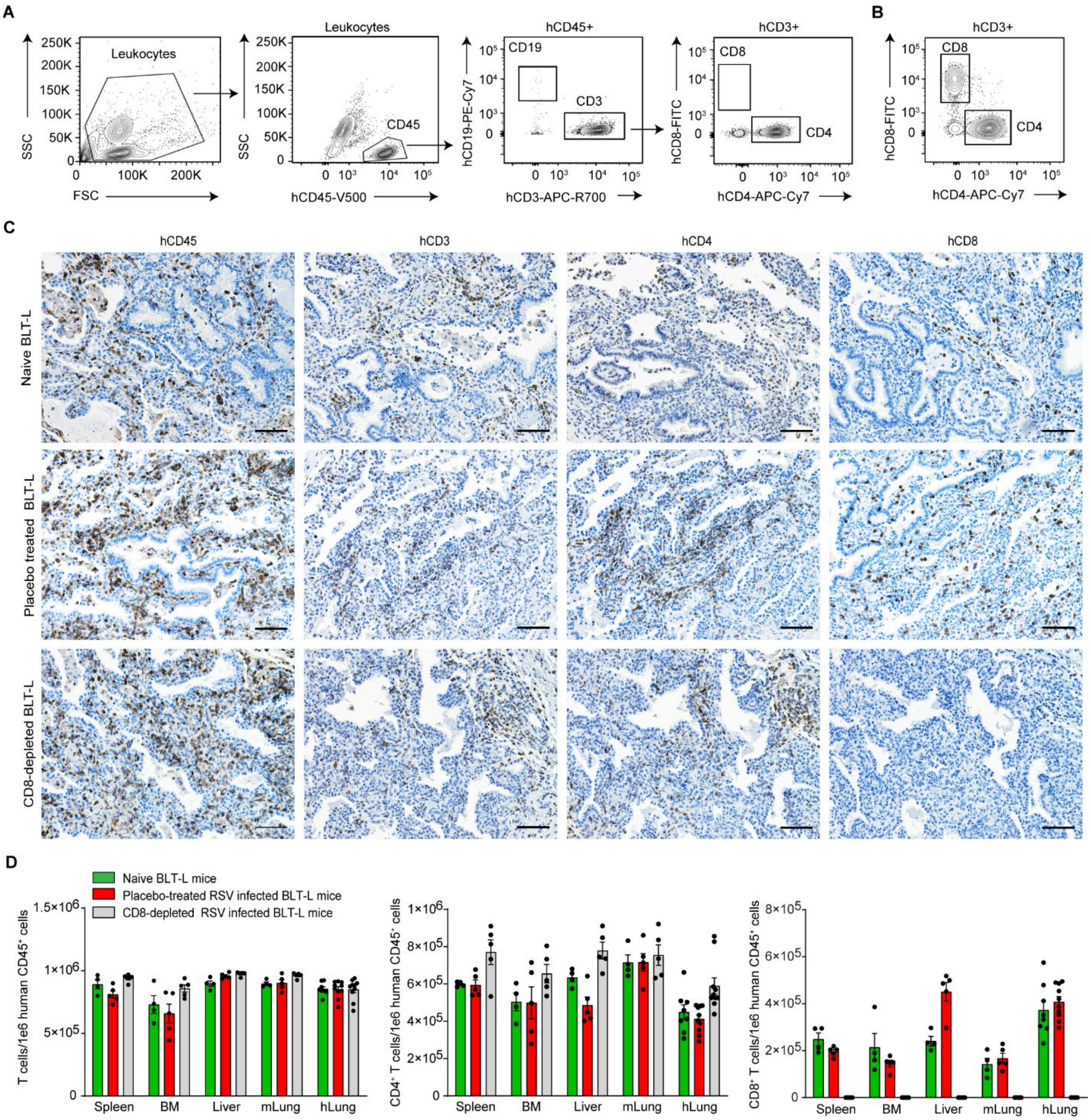

Supplemental Figure 3. Systemic CD8^{+} T cell depletion in RSV-infected BLT-L mice administered CD8-depleting antibody

Flow cytometric gating strategy used for the analysis of CD4^{+} and CD8^{+} T cell levels in BLT-L mice administered (**A**) CD8-depleting antibody or (**B**) placebo. (**C**) Human CD45, CD3, CD4,

and CD8 antigen staining (positive cells, brown; scale bars, 100 um) in human lung implants of naïve BLT-L mice (top panels, n=4 implants analyzed) and RSV-infected BLT-L mice administered placebo (middle panels, n=4 implants analyzed) or CD8-depleting antibody (bottom panels, n=4 implants analyzed). (**D**) Numbers of human T cells, $CD4^+$ T cells, and $CD8^+$ T cells in the spleen, bone marrow (BM), liver, mouse lung (mlung), human lung implants (hlung) of naïve BLT-L (green, n=4 spleen, n=4 BM, n=4 liver, n=4 mlung, n=8 hlung), RSV-infected BLT-L mice (red, n=5 spleen, n=5 BM, n=5 liver, n=5 mlung, n=10 hlung) and RSV-infected CD8 depleted BLT-L mice (gray, n=5 spleen, n=5 BM, n=5 liver, n=5 mlung, n=10 hlung) as determined by flow cytometric analysis. Data shown as mean ± SEM.

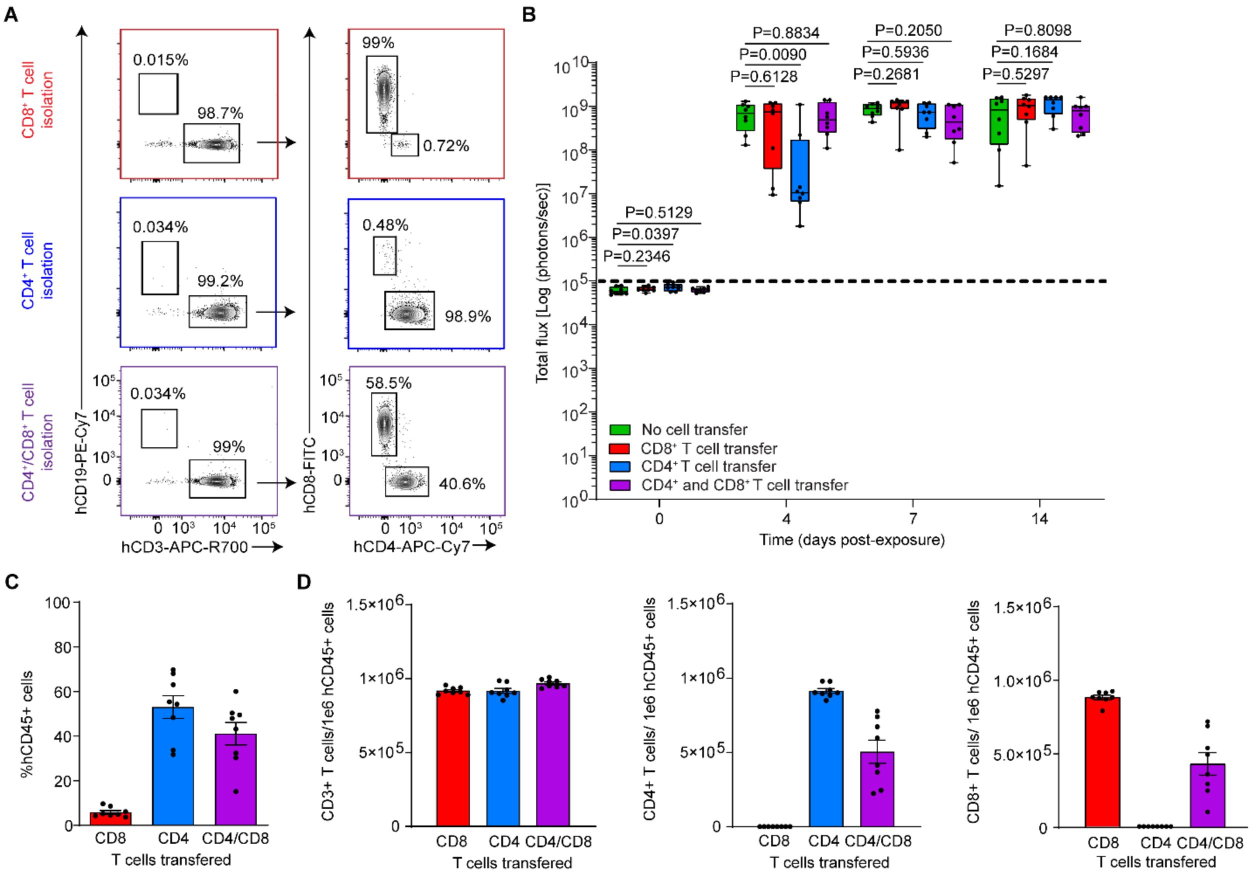

Supplemental Figure 4. Adoptive transfer of human CD4$^+$ and CD8$^+$ T cells isolated from RSV-infected BLT-L mice into RSV-infected LoM

(**A**) Flow cytometry analysis of human CD8$^+$ T cells (red, top panels), CD4$^+$ T cells (blue, middle panels), and CD4$^+$ and CD8$^+$ T cells (purple, bottom panels) isolated from RSV-infected BLT-L mice and transplanted into human donor matched RSV-infected LoM. (**B**) Bioluminescence signal in the human lung implants of LoM (n=8 implants/group) monitored for 14 days following RSV-luc exposure, just prior to adoptive T cell transfer. The median (horizontal line), upper and lower quartiles (box ends), and minimum to maximum values (whiskers) are shown. Statistical significance was determined with a two-tailed Kruskal-Wallis test. *P* values were adjusted for multiple testing using the Benjamini, Krieger, Yekutieli false-discovery rate method. (**C**) Human CD45 levels and (**D**) human T cell (left panel), CD4$^+$ T cell

(middle panel), and CD8$^+$ T cell (right panel) levels in the human lung implants of RSV-infected LoM 10 weeks following adoptive transfer of CD8$^+$ T cells (red bars, n=8 implants), CD4$^+$ T cells (blue bars, n=8 implants), or CD4$^+$ and CD8$^+$ T cells (purple bars, n=8 implants) isolated from RSV-infected BLT-L mice as determined by flow cytometric analysis. Data shown as mean ± SEM. Statistical significance was determined with a two-tailed Kruskal-Wallis test. *P* values were adjusted for multiple testing using the Benjamini, Krieger, Yekutieli false-discovery rate method.

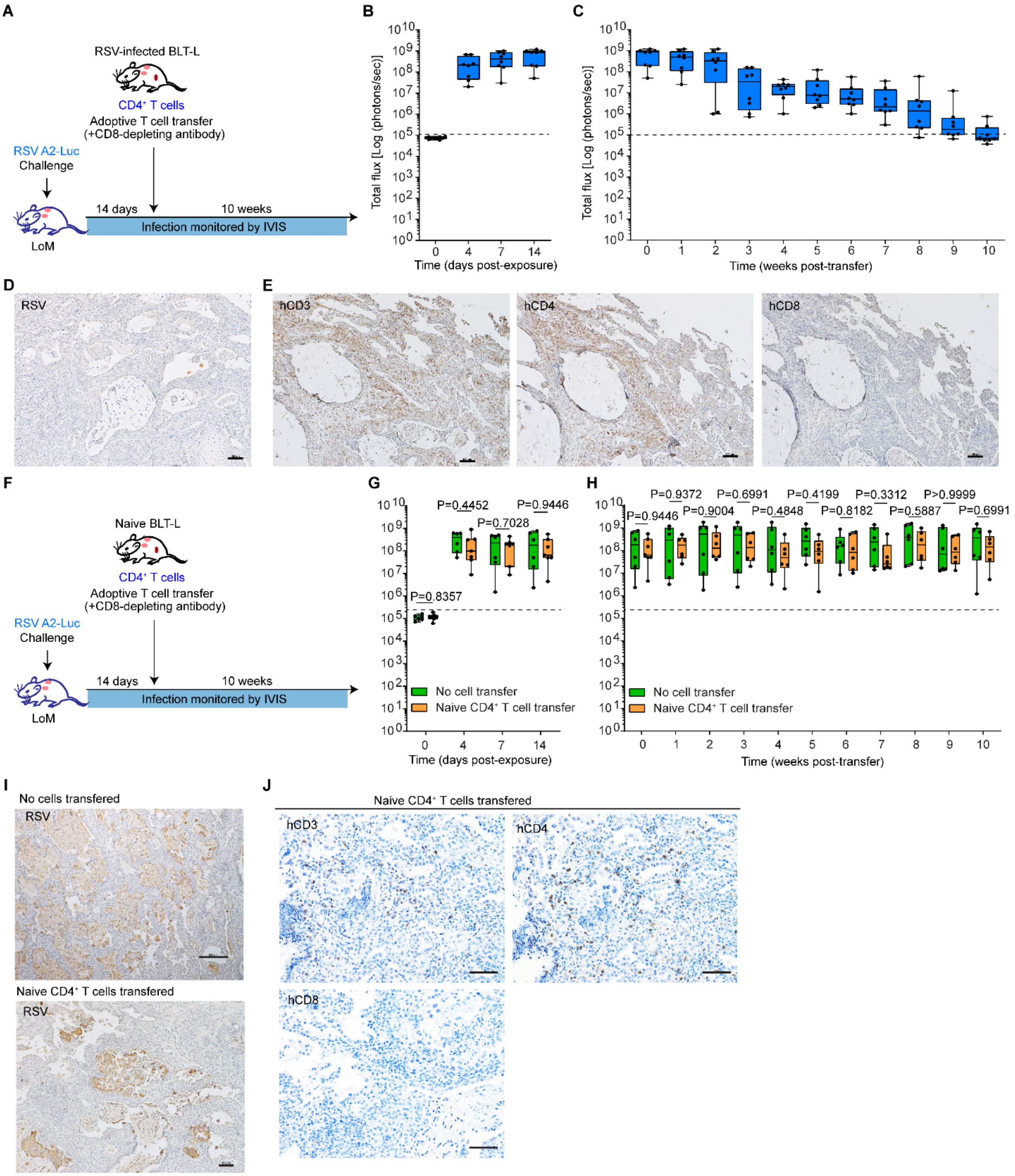

Supplemental Figure 5. Primed CD4$^+$ T cells can control RSV infection *in vivo*

(**A**) LoM were exposed to RSV-Luc and 14 days later transplanted with autologous primed CD4$^+$ T cells (7x10^6) from RSV-infected human donor matched BLT-L mice. RSV replication in

human lung implants of RSV-infected LoM (**B**) prior to and (**C**) post transplantation of primed $CD4^+$ T cells (blue bars, n=8 implants) was monitored by measuring the bioluminescence signal (radiance [p sec^{-1} cm^{-2} sr^{-1}] represented as total flux). (**D**) RSV and (**E**) human CD3, CD4, and CD8 antigen staining (brown; scale bars, 100 um) in lung implants 10 weeks after primed $CD4^+$ T cell transfer (n=4 implants analyzed) to RSV-infected LoM. (**F**) LoM were exposed to RSV-Luc and 14 days later transplanted with autologous unprimed $CD4^+$ T cells ($7x10^6$) from naïve human donor matched BLT-L mice. RSV-infected LoM that did not receive $CD4^+$ T cells served as a control for RSV replication. RSV replication in RSV-infected LoM lung implants (**G**) prior to and (**H**) post transplantation of autologous unprimed $CD4^+$ T cells (orange bars, n=6 implants) or no cells (green bars, n=6 implants) was monitored by measuring the bioluminescence signal (radiance [p sec^{-1} cm^{-2} sr^{-1}] represented as total flux). The median (horizontal line), upper and lower quartiles (box ends), and minimum to maximum values (whiskers) are shown. Statistical significance was determined with a two-tailed Mann-Whitney test. (**I**) RSV-infected cells (brown; left scale bar, 500 um; right scale bar, 100 um); in lung implants 10 weeks after transfer of unprimed $CD4^+$ T cells (n=4 implants analyzed) or no cells (n=4 implants analyzed) to RSV-infected LoM. (J) Human CD3, CD4, and CD8 antigen (brown; scale bars, 100 um) in lung implants 10 weeks after unprimed $CD4^+$ T cell transfer (n=4 implants analyzed) to RSV-infected LoM.
